# Idiopathic desmoid-type fibromatosis of the pancreatic head: case report and literature review

**DOI:** 10.1186/1477-7819-12-103

**Published:** 2014-04-22

**Authors:** Changjun Jia, Baoling Tian, Chaoliu Dai, Xinlu Wang, Xianmin Bu, Feng Xu

**Affiliations:** 1 Department of General Surgery, Shengjing Hospital, China Medical University, 36 Sanhao Street, Shenyang 110004, China; 2 Department of Pathology, Shengjing Hospital, China Medical University, 36 Sanhao Street, Shenyang 110004, China; 3 Department of Ultrasound Medicine, Shengjing Hospital, China Medical University, 36 Sanhao Street, Shenyang 110004, China

**Keywords:** Desmoid-type fibromatosis, Desmoid tumor, Aggressive fibromatosis, Pancreatic head, Musculoaponeurotic tissues, Immunohistochemistry

## Abstract

Desmoid-type fibromatosis (DTF) is an uncommon nonmetastatic fibrous neoplasm. Sporadic intraperitoneal DTF is rarely described in current literature. We herein report a case of DTF of unknown cause involving the pancreatic head. A 41-year-old man presented with recurrent epigastric pain and weight loss. An abdominal computed tomography scan showed a well-delineated solid cystic mass inside the pancreatic head. Pylorus-preserving pancreaticoduodenectomy was performed due to the patient’s debilitating symptoms and suspected malignancy. The pathological examination revealed massive fibroblastic proliferation arising from the musculoaponeurotic tissues, consistent with a diagnosis of DTF. Immunohistochemical phenotyping determined positive immunoreactivity to vimentin and β-catenin, but negative immunoreactivity to smooth muscle actin, CD117, CD34, or S-100, confirming the diagnosis of DTF. No local recurrence or distant metastasis was found during a 24-month follow-up. Radical resection is recommended as first-line treatment for pancreatic DTF. Long-term follow-up studies are required to establish the prognosis of pancreatic DTF.

## Background

Desmoid-type fibromatosis (DTF), also known as desmoid tumor or aggressive fibromatosis, is a rare soft tissue neoplasm. It is locally invasive but without metastatic potential, and is slow-growing [1]. DTFs, which may be extra-abdominal, of the abdominal wall, or intra-abdominal, can arise from any fibrous connective tissues throughout the body [2], but the great majority occur unpredictably at sites of previous trauma, scarring, or irradiation. Moreover, approximately 7.5% of DTF patients have concomitant familial adenomatous polyposis (FAP), especially in intra-abdominal cases [3].

DTF is more frequently reported among the pediatric population (infantile DTF), but can occur at any age. In children, DTFs have primarily been extra-abdominal, involving the skeleton, skeletal muscle, adjacent fascia, aponeurosis, or periosteum. Intra-abdominal DTF (8%) is seen more often in adults, mainly in the gastrointestinal and genitourinary tracts [4]. Although not metastatic, DTF tends to destroy surrounding tissues and organs and recurs frequently after radical resection.

Intra-abdominal DTFs derive primarily from mesenteric connective tissue or the retroperitoneum [5]. These tumors are rarely symptomatic, but encountered incidentally at laparotomy. In addition, an intra-abdominal DTF is more often observed in patients with complicating Gardner’s syndrome or FAP [6]. In some cases, intra-abdominal DTF has been associated with prior abdominal surgery and resembles intraperitoneal recurrence, locoregional metastasis, or abscess following the radical resection of gastrointestinal malignancies [7]. The incidence of sporadic intra-abdominal DTF is low (5%); fewer than 100 cases have been reported since the 1960s. Of these, intra-abdominal DTF of pancreatic origin is particularly rare, with only nine individual cases described in the English literature since the 1980s [6-13] (Table 1).

**Table 1:** **Review of pancreatic desmoid-type fibromatosis (DTF)**^**a**^

**Reference**	**Gender**	**Age (years)**	**Symptoms and signs**	**Lab findings**	**CT features**	**Tumor location**	**Size (cm)**	**Previous surgery**	**Surgery**	**Adjuvant treatment**	**Recurrence**	**Follow-up**
Roggli *et al*. (1980) [6]	M	0.3	Tachypnea, fever, anorexia, weight loss	NA	Solid	Diffuse	NA	None	Biopsy	None	NA	DOD 7 days
Bruce *et al*. (1996) [7]	M	38	Abdominal ache	NA	Solid	Tail	5 × 4.5 × 2.1	Partial pancreatectomy	Resection	NA	No	ANED 24 months
Sedivy *et al*. (2002) [8]	F	68	Weight loss, nausea	Normal	Solid	Head	1.5	Pancreatic biopsy	Resection	NA	NA	NA
Nursal *et a*l. (2003) [9]	F	25	Epigastric pain	NA	Solid	Tail	8.5 × 5.0	None	Biopsy	Symptomatic	NA	NA
Nursal *et al*. (2003) [9]	M	39	Epigastric pain	NA	Solid	Tail	7.5 × 4.0	None	Biopsy	Symptomatic	NA	NA
Pho *et al*. (2005) [10]^a^	M	17	Epigastric pain, weight loss	NA	Cystic	Tail	2.8 × 4.2	None	Resection	Sulindac, tamoxifen, methotrexate, vinblastine	Yes	AWD 24 months
Weiss *et al*. (2006) [11]	M	63	Epigastric pain, abdominal fullness	Normal	Solid	Tail	6.5 × 5.3	Partial pancreatectomy	Resection	None	No	ANED 9 months
Amiot *et al*. (2008) [12]	F	51	Epigastric pain, weight loss	Normal	Cystic	Tail	6	None	Resection	None	No	ANED 12 months
Polistina *et a*l. (2010) [13]	M	68	None	Normal	Cystic	Tail	5	None	Resection	None	No	ANED 60 months
Present study (2013)	M	41	Epigastric pain, weight loss	Normal	Cystic	Head	1.9	None	Resection	None	No	ANED 24 months

^a^All cases were sporadic except for one patient with complicating FAP reported by Pho *et al*. [10]. *CT*, computed tomography; *NA*, not available; *ANED*, alive with no evidence of disease; *AWD*, alive with disease; *DOD*, dead of disease; *NA*, not available.

Herein we report a patient with an isolated, sporadic, and non-trauma-related DTF, located at the pancreatic head and manifesting as a cystic lesion. The patient underwent a successful curative resection and exhibited no signs of recurrence or metastasis within a 24-month follow-up period.

## Review

### Case presentation

A 41-year-old Han Chinese male non-smoker was referred to our surgical unit due to a six-month history of progressive epigastric pain and loss of weight. He denied any radiating pain, nausea, or vomiting. His past medical history showed a healed pulmonary tuberculosis and excision of a neck lipoma, 15 and 10 years previously, respectively. No history of prior abdominal trauma or surgery was noted. The patient had concomitant essential hypertension, but received no antihypertensive treatment. No family history was reported that was suggestive of any genetic disease.

His physical examination was clinically insignificant. The liver function test was normal except for elevations in conjugated and unconjugated bilirubin: total 60.2 μmol/L (reference limits, 3.4 to 20.5 μmol/L), conjugated 19.3 μmol/L (0 to 8.6 μmol/L), and unconjugated 40.9 μmol/L (3.4 to 11.9 μmol/L). The levels of serum tumor markers were within normal limits: carcinoembryonic antigen, 2.5 ng/mL (0 to 5 ng/mL); carbohydrate antigen 19 to 9, 30.3 IU/mL (0 to 37 IU/mL); and α-fetoprotein antigen, 4.2 IU/mL (0 to 9 IU/mL).

**Figure 1:**
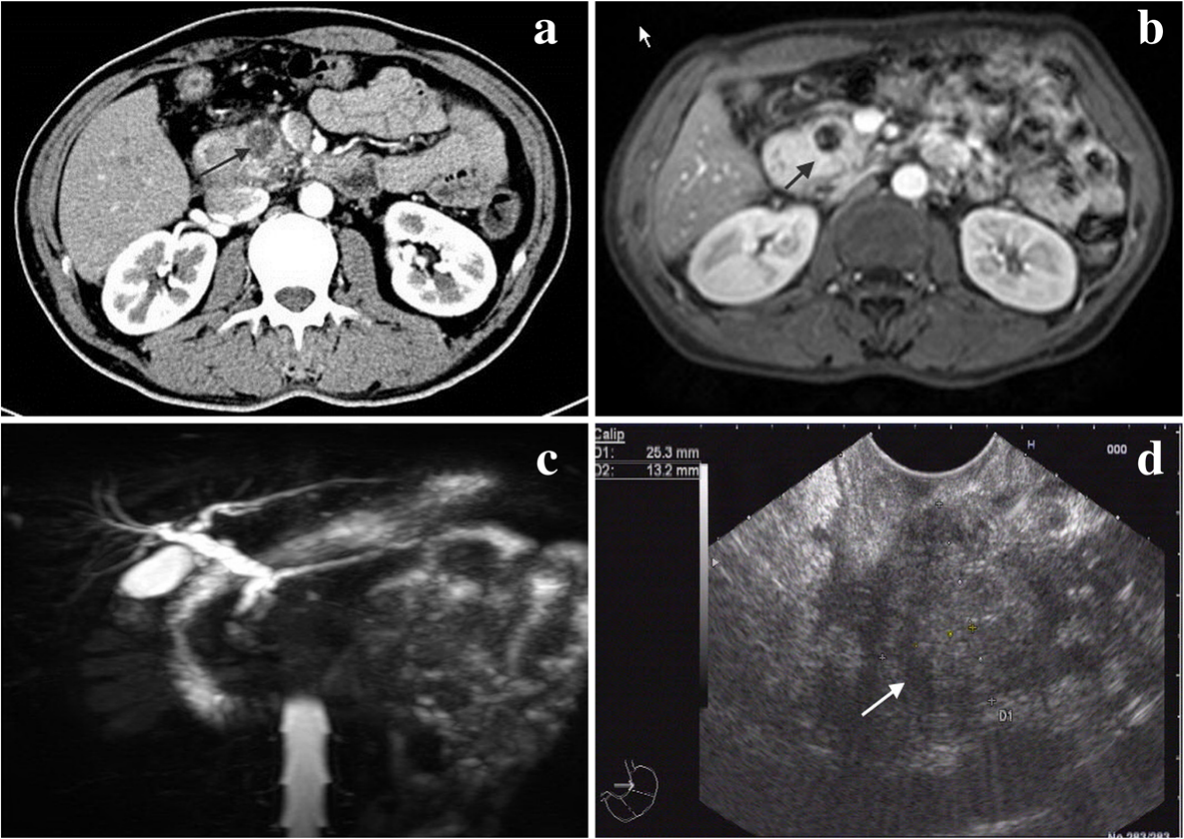
Imaging investigations of the pancreatic head DTF (indicated by the arrow) using (a) abdominal CT scan, (b) MRI scan, (c) MRCP, and (d) EUS.

An abdominal computed tomography (CT) scan revealed a 1.9-cm solid cystic mass in the pancreatic head, with no signs of vascular or visceral invasion (Figure 1a). The pancreatic mass was well-delineated but not encapsulated, with no evidence of local invasion or metastasis. Magnetic resonance imaging (MRI) and endoscopic ultrasonography (EUS) were subsequently ordered to further characterize the anatomical relationship of the pancreatic head mass to the surrounding organs. On MRI scan, a focal cystic mass was found in the uncinate process of the pancreas, showing low signal intensity on T_1_- and T_2_-weighted images (Figure 1b). The peritumoral tissue exhibited a hyperintense shadow in an annular shape on a T_1_-weighted image, whereas this shadow was partially hyperintense without any significant enhancement on a T_2_-weighted image. The descending segment of the duodenum showed a diffuse thickening with potent contrast enhancement on the internal wall and the mucosal lining. Magnetic resonance cholangiopancreatography (MRCP) indicated that the intrahepatic duct, the common bile duct, and the main pancreatic duct were all moderately dilated (Figure 1c). Furthermore, on EUS a well-demarcated, hypoechoic lesion located in the pancreatic head with a normal surrounding region was observed (Figure 1d).

A diagnosis of suspected intraductal papillary mucinous neoplasm or other cystic neoplasm of the pancreas was made for this pancreatic tumor. Elective surgical resection was scheduled as this pancreatic tumor was symptomatic with a suspected diagnosis. During laparotomic exploration, the mass appeared to be localized completely inside the pancreatic head, without any locoregional invasion or lymph node involvement. On gross pathology the tumor appeared as a grayish, dense, cystic, single-cavity mass, free of capsule, without necrosis or hemorrhage. In view of a suspected pancreatic neoplasm, a Traverso-Longmire procedure was performed. The pylorus and spleen were preserved along the lymph node dissection of the peripancreatic region and the celiac axis. The surgical procedure was uneventful and lasted approximately six hours. The volume of intraoperative blood loss was approximately 1000 mL, and the patient received a 400-mL plasma transfusion.

The patient resumed oral intake on postoperative day (POD) 7 but developed an infected intra-abdominal hematoma on POD 13. This condition resolved with antimicrobial therapy as well as peritoneal irrigation and drainage. Oral intake was initiated on POD 18, and the patient was discharged from the hospital on POD 21.

**Figure 2:**
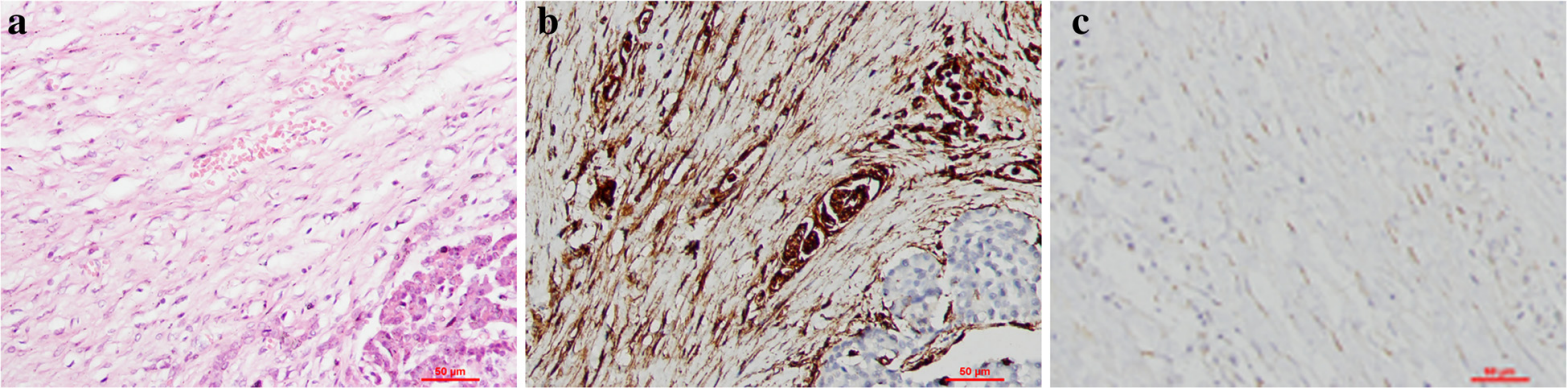
Histopathology of the pancreatic head DTF: (a) histology (200×, scale bar = 50 μm) and (b, c) immunohistochemistry (200×, scale bar = 50 μm) showing positive immunoreactivity against (b) vimentin and (c) β-catenin.

The histological examination confirmed a clean resection margin. Histological sections showed a large number of spindle-shaped cells, with a regular nuclear pattern within a background of massive collagen fibers. The peritumoral parenchymal tissue was edematous and infiltrated by tumor cells and a small number of inflammatory cells (Figure 2a). No marked cell death or mitosis was observed inside the tumor tissue. The cystic component was found to be a dilated pancreatic duct filled with pancreatic secretion. The duodenal muscularis propria, the locoregional lymph nodes, and the pancreatic plexus were tumor-free.

Further immunohistochemical analysis was ordered to differentiate uncommon gastrointestinal tumors of mesenchymal or neural origin; that is, DTF, fibrosarcoma, gastrointestinal stromal tumor, and neurosarcoma. The analysis revealed that the tumor cells were immunopositive for vimentin (Figure 2b) and β-catenin (Figure 2c), but immunonegative for smooth muscle actin, S-100, CD117, and CD34. These pathological features were consistent with a diagnosis of intra-abdominal DTF [3].

The patient received no adjuvant therapy after surgery. He was followed-up regularly at the outpatient clinic using routine hematological and biochemical tests, as well as abdominal CT scan. He exhibited good health and complained of no discomfort. The total bilirubin level returned to normal after the resection. No signs of local recurrence or distant metastases were identifiable on abdominal CT scan by the last scheduled follow-up visit at 24 postoperative months.

## Discussion

Pancreatic tumors are usually (approximately 95%) primary cancers of exocrine origin, namely, adenocarcinomas; the remainder arise from neuroendocrine cells. Pancreatic DTFs are extremely rare and encountered incidentally in surgical practice. Only nine previous cases of pancreatic DTFs have been reported within the last three decades in the English medical literature [6-13] (Table 1). Of these nine cases, all occurred in middle-aged patients except for one infant [6]. Three patients had cystic pancreatic tumors and one patient had concomitant FAP. Seven of the pancreatic DTFs were located in the pancreatic tail, one in the head, and one case was diffuse throughout the pancreas. To the best of our knowledge, our patient is the second reported case of DTF of the pancreatic head in the English literature.

The etiology of intra-abdominal DTF is unknown. The risk factors include being female, genetic mutation, positive family history, and previous history of abdominal surgery. Because women are more prone to sporadic intra-abdominal DTF than men, (a female-to-male ratio of 2:1 to 5:1), estrogen might have a pathogenetic role [14]. However, for diagnosed pancreatic DTF in particular, the literature review and our report identified seven males and three females.

FAP is frequently associated with intra-abdominal DTF, but only one pancreatic DTF patient was reported to have concomitant FAP in the current literature [10]. Two siblings harbored pancreatic and pelvic DTFs in the absence of FAP [9].

A somatic mutation in the genes 3′-adenomatous polyposis coli (*APC*) or β-catenin (*CTNNB1*) is the most significant risk factor for intra-abdominal DTF in FAP patients [15,16]. The protein APC assists in the regulation of cellular β-catenin, which is implicated in the process of wound healing and fibroproliferation. APC and β-catenin are both members of the Wnt signaling pathway, which may be aberrantly expressed in DTF, irrespective of familial or sporadic origin [17]. The translocation of cytoplasmic β-catenin into the nucleus activates the expression of T-cell factor, which in turn upregulates the expressions of downstream genes such as cytochrome oxidase 2 (*COX2*), platelet-derived growth factor (*PDGF*), matrix metallopeptidase (*MMP*), hyaluronan-mediated motility receptor (*RHAMM*), and retinoblastoma 1 (*RB1*) [18]. These genetic mechanistic factors may underlie the variable biological behavior and clinical course of DTF. No specific genetic study has investigated such susceptibility in pancreatic DTF, as only a few individual cases have been reported.

Previous abdominal surgery is said to initiate the proliferation of fibrous tissue and is therefore implicated in the occurrence and recurrence of intra-abdominal DTF. However, of the reported pancreatic DTF patients, only two had undergone previous abdominal surgery, one of whom developed a sporadic DTF at the pancreatic suture line [7], and the other a DTF of the pancreatic stump following a distal pancreatectomy for pancreatic neuroendocrine tumors [11]. Our case is sporadic and not associated with FAP or abdominal surgery.

DTF is characterized by a highly variable and unpredictable clinical course of tumor growth, stabilization, and occasionally even spontaneous regression in the absence of medical intervention [19]. DTF-associated symptoms depend on the location of the tumor. Intra-abdominal DTF is usually asymptomatic or symptoms are non-specific, such as abdominal discomfort or pain and weight loss. If blood vessels, the gastrointestinal tract, or the urinary tract is involved, a patient may present with symptoms of compression or obstruction. Like pancreatic cancer, pancreatic DTF is usually silent in terms of clinical signs. Epigastric pain, the most common complaint, seldom radiates to the back as in pancreatic cancer. Weight loss is also often seen in pancreatic DTF patients due to chronic aversion to food. Painless jaundice, a classic manifestation of pancreatic head cancer, is rarely seen in patients with pancreatic head DTF as it usually does not obstruct the common bile duct [8]. However, our patient had a mild elevation in bilirubin which returned to normal after radical resection. In most cases, laboratory findings and levels of serum tumor markers stay within normal limits.

A confirmed diagnosis of sporadic intra-abdominal DTF prior to surgery is very unlikely, while a tentative diagnosis is mainly based on clinical suspicion combined with medical imaging evaluation. A history of FAP may raise the possibility of intra-abdominal DTF, although pancreatic DTF is usually sporadic. Medical imaging evaluation is important for characterizing the location of pancreatic DTF in relation to the surrounding tissues and organs (including the pylorus, duodenum, common bile duct, and spleen) and for determining its resectability. On imaging studies, pancreatic DTF usually manifests as a well-defined solid or solid cystic lesion. On ultrasonography, it is homogeneous and hypoechoic; on CT scan, it appears as hypodense soft tissue; and on MRI, signal intensity is low. The tumor is expected to be mildly enhanced in the presence of contrast media, as it consists essentially of over-proliferation of fibrous tissue. EUS is very useful for visually defining the pancreatic tumor located in the pancreatic head, and to determine whether the major vessels and the duodenum are infiltrated by the tumor. Additionally, EUS allows fine-needle aspiration biopsy for cytological examination of superficially located tumors [20,21]. In the present case it was difficult to differentiate pancreatic DTF from pancreatic cystadenocarcinoma on CT/MRI scan, although contrast images suggested the presence of a primary pancreatic tumor.

The typical histology of DTF shows regular fibroblasts and fibrocytes in a well-oriented pattern, but also with an infiltrative growth pattern within a background of massive collagen bundles. However, it is histologically challenging to differentiate pancreatic DTF from other uncommon pancreatic tumors such as low-grade fibrosarcoma, neuroendocrine tumor, and gastrointestinal stromal tumor. DTF is sometimes thought to be a potential sarcoma due to its aggressive growth and local invasion, but pathologic mitosis or metastasis is very unlikely [22].

Immunohistochemistry against specific cell markers of various origins is a very effective diagnostic tool for the differentiation of pancreatic tumors. Pancreatic DTF expresses the markers for mesenchymal cells (vimentin) but not markers for stromal cells (CD117 and CD34) or neural cells (S-100). β-catenin immunohistochemistry is reported to be useful for distinguishing deep DTF from other benign or malignant fibroblastic and myofibroblastic lesions [22]. Thus, the pathological diagnosis of pancreatic DTF is established and that of pancreatic gastrointestinal stromal tumor can be excluded, based on the histological and immunohistochemical findings.

Multimodal therapy has been reported for the treatment of intra-abdominal DTF, including pancreatic DTF. Watching and waiting is an acceptable option for patients with asymptomatic or minimally symptomatic DTF, as it is a non-metastatic and slow-growing tumor. However, surgical intervention seemed mandatory in our case as the presence of pancreatic DTF was seriously compromising the quality of life of this patient, and its obscure nature made it potentially life-threatening.

The mainstay of pancreatic DTF treatment is radical resection. In most previous reports, patients underwent distal pancreatectomy or pancreaticoduodenectomy, except for three patients who received only a tumor biopsy. The primary concern of the physician regarding surgical resection is the high likelihood of local recurrence: the reported recurrence rate of DTF after resection is 19 to 77% [23], and is higher in patients with complicating FAP or Gardner’s syndrome. A recurrent DTF may be resectable but is associated with a greater surgical morbidity as a second-look surgery is performed. A clean resection margin contributes favorably to a reduced recurrence rate, as shown by clinicopathological data [24].

Alternative or adjuvant therapies have also been proposed for the management of intra-abdominal DTF. Non-steroidal anti-inflammatory drugs (NSAIDs) were reported to successfully downsize a pelvic DTF in a man with complicating lower limb vascular compression [25]. Hormonal therapy using non-steroidal benzothiophene (tamoxifen or raloxifen) effectively obtained a complete or partial response in 13 intra-abdominal DTF patients with complicating FAP [26]. Other treatment options include chemotherapy (vinblastine, methotrexate, doxorubicin, and dacarbazine) [27], interferon [28], and irradiation therapy [29]. These modalities are mainly used if radical resection is determined to be unfeasible or mutilating. Imatinib, a targeted receptor tyrosine kinase inhibitor used for treating gastrointestinal stromal tumor, has also been attempted for the treatment of DTF after multiple cycles of chemotherapy [30]. In current literature, only one pancreatic DTF patient with complicating FAP received NSAID and tamoxifen, as well as chemotherapy with methotrexate and vinblastine, in a case of local recurrence following surgical resection [10]. This suggests that curative resection may be adequate for the treatment of pancreatic DTF if not complicated with FAP.

The prognosis of pancreatic DTF is not known. Recurrence after curative resection has not been observed during mid- and long-term follow-up, except for a single patient with a congenital generalized fibromatosis [10]. A favorable survival outcome has been shown in most cases - almost 100% for overall survival and > 80% for progression-free survival at five years [31]. Severe pain or narcotic dependency, tumor size > 10 cm, and need for total parenteral nutrition are negatively associated with survival [32]. Most reported pancreatic DTF patients survived, disease-free. Our patient has been followed-up for 24 postoperative months and remains free of disease at the preparation of this manuscript, consistent with previous reports.

## Conclusion

In conclusion, sporadic pancreatic DTF is a rare non-metastatic soft tissue tumor that can be locally quite destructive, and in our case, debilitating. Initial diagnosis is mainly based on clinical suspicion and medical imaging. The diagnosis is confirmed via immunohistochemistry to differentiate DTF from other uncommon soft tissue tumors originating from the pancreas. Radical resection with a clean margin is recommended as the first-line treatment for DTF, although FAP patients are more prone to recurrence following surgical resection. Adjuvant therapies may be effective for unresectable or recurrent DTF. Long-term prognosis is currently unknown, and a regular follow-up is essential.

## Abbreviations

DTF: desmoid-type fibromatosis; FAP: familial adenomatous polyposis; CT: computed tomography; MRI: Magnetic resonance imaging; EUS: endoscopic ultrasonography; MRCP: magnetic resonance cholangiopancreatography; POD: postoperative day; NSAIDs: non-steroidal anti-inflammatory drugs.

## Competing interests

The authors declare that they have no competing interests related to the publication of this report.

## Authors’ contributions

CJ and XW prepared the manuscript and conducted the literature search; CD reviewed and edited the manuscript; XB corrected and revised the manuscript; CD, CJ, and FX treated and observed the patient; XW provided the radiographic and ultrasound images; BT performed the histopathological and immunohistochemical examinations. All authors read and approved the final manuscript.
